# Inhibition of Adaptive Immune Responses Leads to a Fatal Clinical Outcome in SIV-Infected Pigtailed Macaques but Not Vervet African Green Monkeys

**DOI:** 10.1371/journal.ppat.1000691

**Published:** 2009-12-11

**Authors:** Jörn E. Schmitz, Roland C. Zahn, Charles R. Brown, Melisa D. Rett, Ming Li, Haili Tang, Sarah Pryputniewicz, Russell A. Byrum, Amitinder Kaur, David C. Montefiori, Jonathan S. Allan, Simoy Goldstein, Vanessa M. Hirsch

**Affiliations:** 1 Division of Viral Pathogenesis, Beth Israel Deaconess Medical Center, Harvard Medical School, Boston, Massachusetts, United States of America; 2 Laboratory of Molecular Microbiology, National Institute of Allergy and Infectious Diseases, National Institutes of Health, Maryland, United States of America; 3 Laboratory for AIDS Vaccine Research and Development, Department of Surgery, Duke University Medical Center, Durham, North Carolina, United States of America; 4 Division of Immunology, New England Primate Research Center, Southborough, Massachusetts, United States of America; 5 Division of Clinical Research, National Institute of Allergy and Infectious Diseases, National Institutes of Health, Bethesda, Maryland, United States of America; 6 Department of Virology and Immunology, Southwest Foundation for Biomedical Research, San Antonio, Texas, United States of America; SAIC-Frederick, United States of America

## Abstract

African green monkeys (AGM) and other natural hosts for simian immunodeficiency virus (SIV) do not develop an AIDS-like disease following SIV infection. To evaluate differences in the role of SIV-specific adaptive immune responses between natural and nonnatural hosts, we used SIV_agmVer90_ to infect vervet AGM and pigtailed macaques (PTM). This infection results in robust viral replication in both vervet AGM and pigtailed macaques (PTM) but only induces AIDS in the latter species. We delayed the development of adaptive immune responses through combined administration of anti-CD8 and anti-CD20 lymphocyte-depleting antibodies during primary infection of PTM (n = 4) and AGM (n = 4), and compared these animals to historical controls infected with the same virus. Lymphocyte depletion resulted in a 1-log increase in primary viremia and a 4-log increase in post-acute viremia in PTM. Three of the four PTM had to be euthanized within 6 weeks of inoculation due to massive CMV reactivation and disease. In contrast, all four lymphocyte-depleted AGM remained healthy. The lymphocyte-depleted AGM showed only a trend toward a prolongation in peak viremia but the groups were indistinguishable during chronic infection. These data show that adaptive immune responses are critical for controlling disease progression in pathogenic SIV infection in PTM. However, the maintenance of a disease-free course of SIV infection in AGM likely depends on a number of mechanisms including non-adaptive immune mechanisms.

## Author Summary

Simian immunodeficiency virus (SIV) is a naturally occurring infection in a wide range of African nonhuman primates, including African green monkeys (AGM), which generally results in a clinically inapparent infection. In contrast, SIV infection of Asian nonhuman primates such as macaques can result in an AIDS-like disease similar to that observed in humans infected with human immunodeficiency virus (HIV). This different pathogenic outcome occurs despite similar levels of viremia. In order to evaluate the contribution of adaptive immune responses to these different outcomes, we transiently inhibited the generation of CD8+ and CD20+ lymphocyte-mediated immune responses in vervet AGM and pigtailed macaques (PTM) during primary SIV infection. PTM experienced higher viremia and accelerated progression to disease, whereas AGM showed only a short prolongation of peak viremia but exhibited no signs of illness. These results demonstrate that protection against development of disease in AGM does not solely rely on adaptive immune responses. Future efforts should aim to determine the underlying mechanisms that enable natural hosts to cope with SIV infection and to apply these findings to develop new treatment modalities for humans infected with HIV.

## Introduction

Although it is not known when SIV was first introduced into African nonhuman primates, it is widely believed that African monkey and ape species coevolved with SIV infection probably for tens of thousands of years [1],[2]. In contrast, Asian nonhuman primates and humans encountered the virus much more recently [2],[3]. Despite these differences, SIV infections in nonhuman primates and HIV in humans follow a similar pattern of viremia: an initial burst of viremia during primary infection followed by a partial containment and establishment of a plateau or set point viremia [4],[5],[6]. Additionally, the level of viremia in African monkeys, natural hosts of SIV, and Asian monkeys, nonnatural hosts of SIV infection is similar [7],[8],[9],[10]. Given the similarities in viral load, however, the course of infection and its consequences differ between natural and nonnatural hosts [11],[12],[13]. Most natural hosts such as AGM appear to peacefully coexist with the SIV infection while macaques generally develop overt signs of illness, immune failure and AIDS [14]. However, recent findings indicate that some natural hosts like chimpanzees may develop an AIDS-like disease when infected with SIV [15].

These differences in pathogenic consequences of infection prompt speculation about the mechanisms that enable African primate species to cope with SIV infection without developing disease. AGM provide a dramatic contrast to the apparently irrevocable pathway to immune failure seen in SIV-infected macaques and HIV-infected humans. At least two fundamental characteristics of SIV infection of natural host species that appear to distinguish them from pathogenic infections include the lack of chronic immune activation and the paucity of CCR5+ CD4+ target cells [11],[16],[17]. These differences suggest that natural hosts may have developed a complex arsenal of protective mechanisms to cope with the pathogenic consequences of SIV-infection. Adaptive immune responses, such as SIV-specific CD8+ T cells and humoral immune responses, have also been observed in SIV-infected natural hosts either at comparable or lower magnitude than in pathogenic SIV and HIV infection [18],[19],[20],[21],[22],[23],[24]. However, the ultimate role of adaptive immune responses in the protection against disease progression in AGM and other natural hosts of SIV remain elusive.

An ideal setting to study the role of adaptive immune responses is to utilize the same virus strain of SIV in two different host species that would respond with similar dynamics of viremia but disparate disease outcome. Previously, it was shown that SIV_agmVer90_ can induce AIDS in pigtailed macaques (*Macaca nemestrina*) but not in vervet AGM (*Chlorocebus pygerythrus*) [25]. In fact, SIV_agmVer90_ infection induces an AIDS-like disease in PTM, similar to that observed in SIV_mac251_-infection in rhesus monkeys. Critically for the present study, set point viremia in SIV_agmVer90_-infection of PTM and AGM is similar. This observation confirms a characteristic finding in natural hosts: disparate pathogenic outcomes despite a similar magnitude of viremia as seen in nonnatural hosts. In fact, in natural hosts the magnitude of viremia varies widely, without any clinical consequences [7]. In contrast, the extent of viremia is an excellent predictor of disease progression in pathogenic models such as macaques and humans [26],[27],[28].

Here, we utilized the administration of antibodies to deplete both CD8+ lymphocytes and B cells during primary SIV_agmVer90_ infection in AGM and PTM to delay cellular and humoral SIV-specific immune responses. These studies underlined the critical role of adaptive immune responses in viral control in nonnatural hosts like PTM. In contrast, the absence of clinical signs of disease in AGM suggested that the maintenance of a disease-free course of infection in natural hosts does not solely depend on adaptive immune responses.

## Results

### Depletion of CD8+ and CD20+ lymphocytes in vervet AGM and PTM

To better understand the role of adaptive immune responses during the early stages of SIV infection in pathogenic and non-pathogenic models of SIV infection, we initiated the depletion of CD8+ and CD20+ lymphocytes prior to infection with SIV. Six vervet AGM and six PTM received five doses of humanized anti-CD8α cM-T807 monoclonal antibody (mAb) on days 0, 3, 7, 10 and 14 and three doses of anti-CD20 human mAb on days −7, 14 and 35. Four lymphocyte-depleted animals of each species were also inoculated intravenously with SIV_agmVer90_ [25] on day 0, and two AGM and PTM served as uninfected controls.

**Figure 1:**
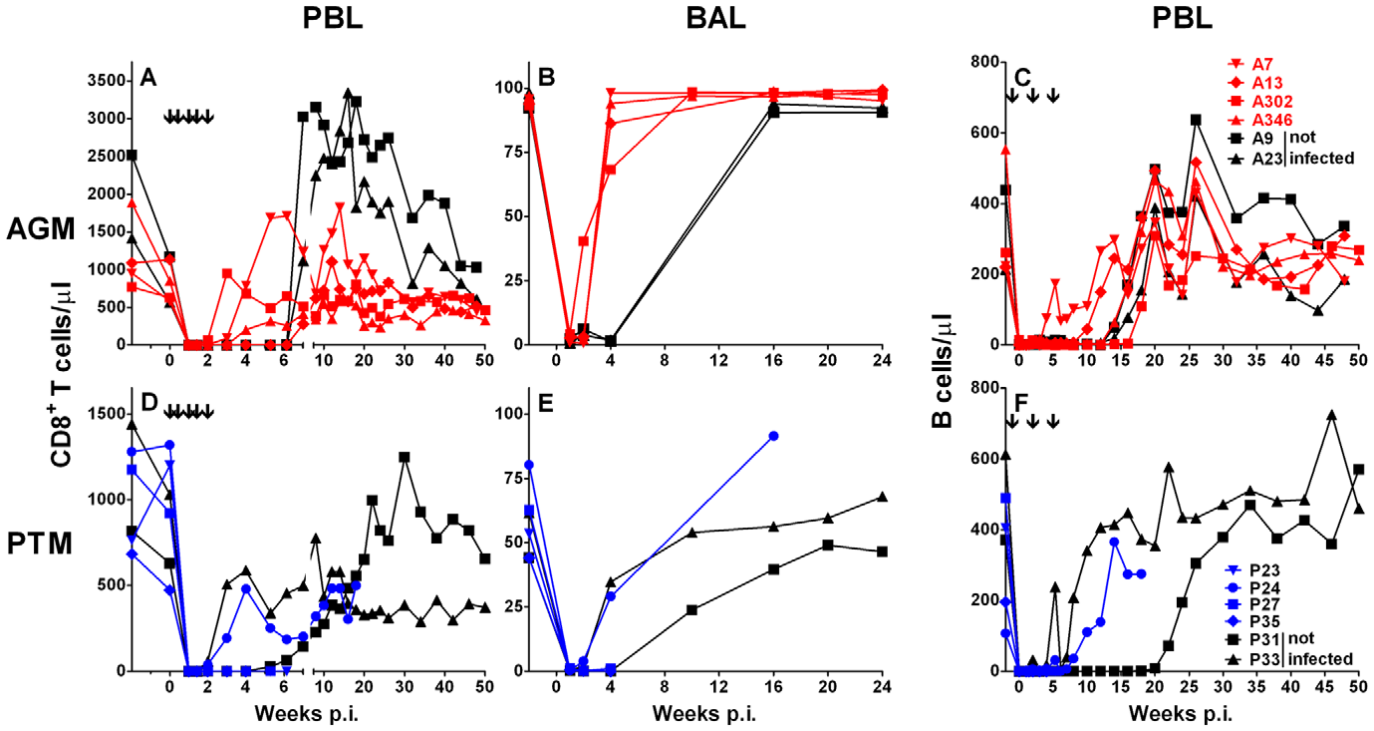
CD8+ T cell and CD20+ B cell depletion in AGM and PTM. Anti-CD8 antibody administration-induced depletion of CD8+ lymphocytes in peripheral blood lymphocytes (PBL) and bronchoalveolar lavage (BAL) in AGM (**A, B**) and PTM (**D, E**). Anti-CD20 antibody administration induced depletion of B cells in PBL of vervet AGM (**C**) and of PTM (**F**). Animals that received anti-CD8 and anti-CD20 antibodies but were not inoculated with SIV are shown with black symbols. Animals that received anti-CD8 and anti-CD20 antibodies and were inoculated with SIV_agmVer90_ are shown in color symbols (red for AGM, blue for PTM). The black arrows in panels **A** and **D** indicate the injection of the anti-CD8α mAb cM-T807. The black arrows in panels **C** and **F** indicate the injection of the anti-CD20 mAb Rituximab.

**Figure 2:**
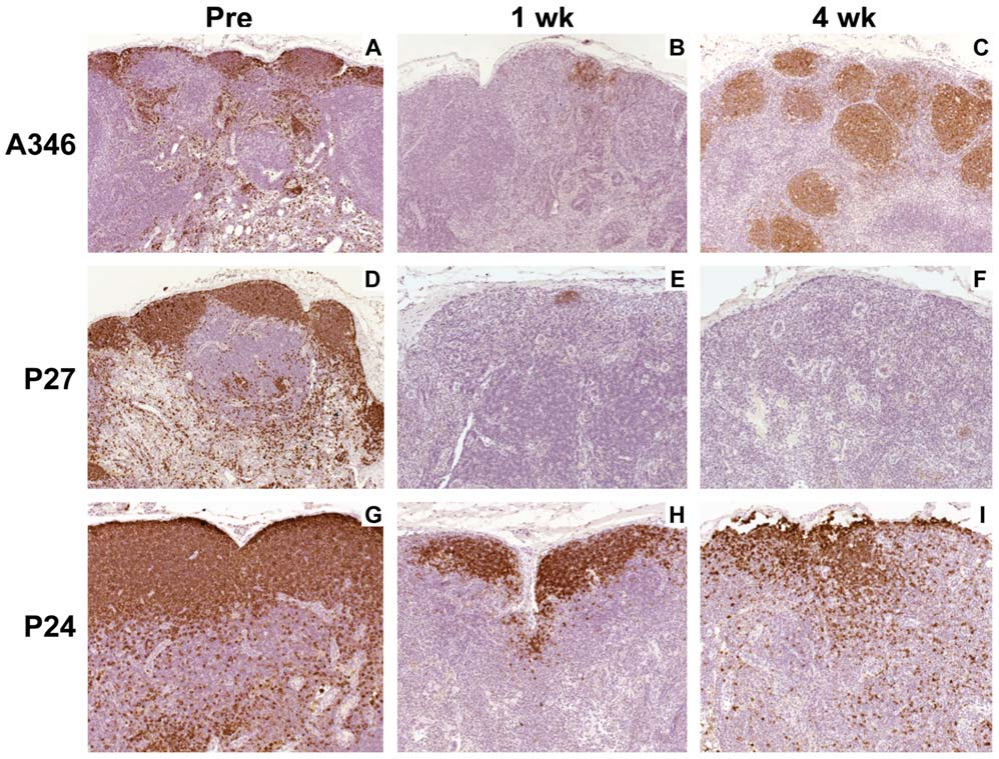
Only partial depletion of B cells in lymphatic tissues of CD8+ and CD20+ lymphocyte-depleted AGM but not of PTM. Immunohistochemical (IHC) detection of CD20+ B cells in sequential lymph node biopsies from a representative vervet AGM (A346) collected pre-inoculation (**A**), one week p.i. (**B**), and four weeks p.i. (**C**). Representative IHC staining for CD20 is shown on two PTM with different clinical courses. PTM P27 was representative of the pattern seen in the three PTM that showed efficient depletion of CD20+ B cells at one week p.i. that was irreversible at four weeks p.i. (**D, E, and F**). PTM P24 only had a partial depletion of CD20+ B cells at one week p.i. and near normal CD20+ B cell levels at four weeks p.i. (**G, H, and I**).

The treatment with lymphocyte depleting mAb resulted in a transient depletion of both CD8+ T cells and CD20+ B cells from peripheral blood in the AGM (**Fig. 1A and C**). A comparable period of lymphocyte depletion, 6 and 14 weeks for CD8+ T cells and B cells, respectively, was also observed in the uninfected control AGM (A9 and A23). The SIV-infected AGM had a median CD8+ T cell depletion of 3.5 weeks (range: 2–6 weeks) and a median B cell depletion of 12 weeks (range: 4–18 weeks). AGM A7 showed the earliest resurgence of both CD8+ T and B cells (3 and 4 weeks, respectively). Vervet AGM harbor two distinct subsets of CD8+ T cells: CD8αα homodimer and CD8αβ heterodimer expressing cells [18]. Despite a lower expression of the CD8 molecule on CD8αα T cells, administration of the cM-T807 mAb affected both CD8+ T cell subsets (data not shown). The cells that first reappeared after CD8+ T cell depletion were mainly CD8αα+ T cells. Since depletion of peripheral blood lymphocyte subsets does not reflect depletion in tissues, we performed flow cytometric analyses for CD8+ and CD20+ lymphocyte subsets in bronchoalveolar lavage samples (BAL) and lymph node biopsies. A shorter duration of CD8+ T cell depletion was observed in BAL and lymph nodes of SIV-infected AGM where CD8+ T cells rebounded at week three (**Fig. 1B** and data not shown). This was associated with a transient increase in double negative (CD4− CD8−) T cells in BAL consistent with down-modulation or masking of the CD8 molecule on T cells (data not shown). The SIV negative control animals were CD8+ lymphocyte-depleted in BAL and lymph nodes throughout week four post depletion. Four of the lymphocyte-depleted AGM had a long lasting depletion of B cells in peripheral blood for 13–16 weeks and two AGM (A7 and A13) had a shorter depletion of 4 and 10 weeks post infection (p.i.) (**Fig. 1C**). A similar transient depletion of B cells was also observed in lymph node sections at one and four weeks p.i. (data not shown and **Fig. 2A–C**).

Four of the six lymphocyte-depleted PTM showed an efficient and long lasting depletion of CD8+ T cells and B cells (P31, P23, P27, P35) (**Fig. 1D, E and F**). Depletion of both CD8+ T cells and B cells was irreversible in three of the SIV-inoculated PTM (P23, P27, and P35). Each of these PTM rapidly developed disease and had to be euthanized by 6–7 weeks. Transient depletion was observed in one of the SIV-infected PTM, P24, and one uninfected animal, P33. Both PTM had a depletion of CD8+ T cells for two weeks and a partial depletion of CD20 cells with a reappearance of B cells at week 8 post depletion (**Fig. 1D and F**). The uninfected PTM P31 had a long lasting B cell depletion of 20 weeks and a CD8+ T cell depletion of 5 weeks. Analysis of CD20 expression in peripheral lymph node sections correlated with the flow cytometric analysis of peripheral blood. Depletion of B cells in the long-term depleted PTM was very efficient in lymph node sections (**Fig. 2D–F**). This contrasted with incomplete depletion in lymph node biopsies collected at one and four weeks post depletion from P24 (**Fig. 2G–I**), the one SIV-infected PTM with incomplete peripheral depletion.

### CD8+ T cell and CD20+ lymphocyte depletion increases peak viremia in PTM but not AGM

**Figure 3:**
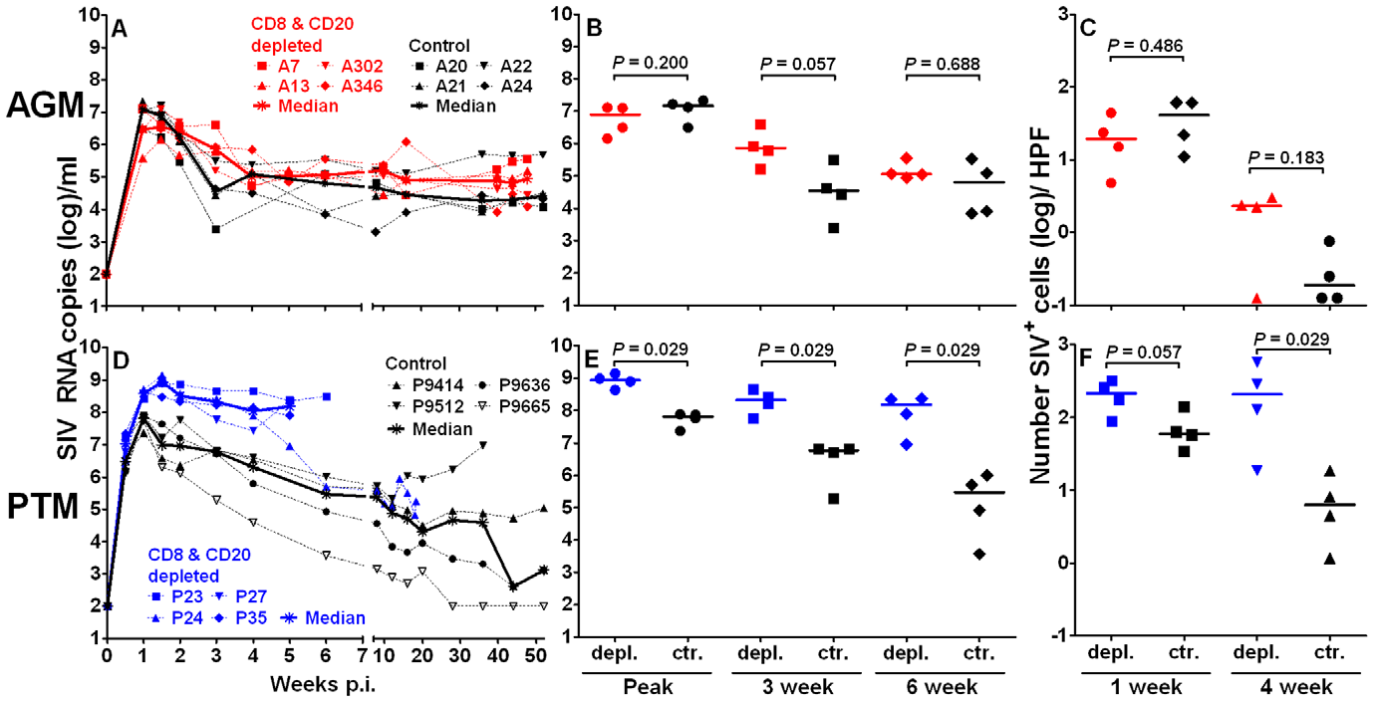
CD8+ and CD20+ lymphocyte depletion has a greater effect on SIV peak viremia and lymphatic tissue viral load in PTM than in AGM. Plasma SIV RNA copies/ml are shown for vervet AGM (**A, B**) and for PTM (**D, E**). Number of SIV RNA expressing cells in lymph node biopsies of anti-CD8 and anti-CD20 treated and historic control AGM (**C**) and PTM (**F**) at one and four weeks p.i. The median number of SIV positive cells is given for each animal and was quantified for six high power fields (HPF). CD8+ and CD20+ lymphocyte-depleted animals are plotted in color symbols (red symbols for vervet AGM and blue symbols for PTM) and historic control animals in black symbols.

**Figure 4:**
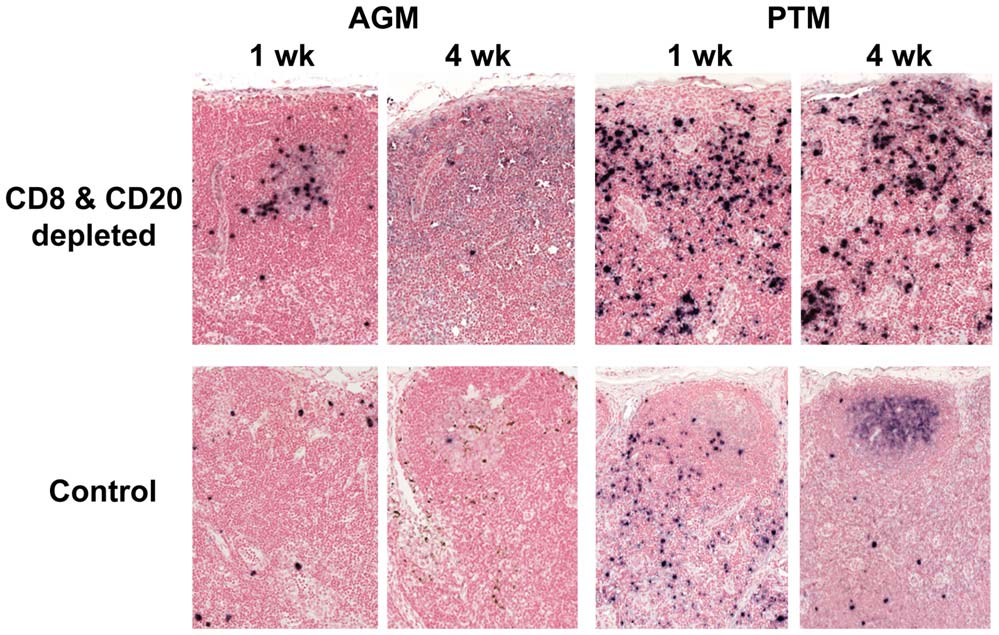
CD8+ and CD20+ lymphocyte depletion in SIV_agmVer90_-infected PTM results in enhanced virus replication in contrast to AGM. Representative SIV-specific *in situ* hybridization of lymph nodes collected at one and four weeks p.i. from a vervet AGM (left) and PTM (right). Top panels show samples from CD8+ and CD20+ lymphocyte-depleted animals and bottom panels show samples from historical control animals. Active viral replication in SIV positive cells is marked in black color.

CD8+ and CD20+ lymphocyte depletion had only a brief effect on the plasma RNA viral load in AGM. The viral load followed a course comparable to that observed in SIV-infected historical control AGM that were inoculated with the same virus (**Fig. 3A**). The peak SIV viral load was similar between CD8+ and CD20+ lymphocyte-depleted AGM and control AGM (median: 0.79×10^7^ SIV RNA copies/ml in the depleted AGM versus 1.47×10^7^ SIV RNA copies/ml in the control AGM; *P* = 0.200 **Fig. 3B**). There was however a trend towards a prolongation of peak viremia in the CD8+ and CD20+ lymphocyte depleted AGM. The median viral load was higher at three weeks p.i. in the antibody-treated AGM than in the historic controls (lymphocyte-depleted: 7.22×10^5^ SIV RNA copies/ml versus control: 0.35×10^5^ SIV RNA copies/ml; *P* = 0.057). This trend was not maintained and differences in viremia between the two groups of AGM vanished at week six p.i. (median: 1.14×10^5^ SIV RNA copies/ml versus 0.645×10^5^ SIV RNA copies/ml; *P* = 0.688). To confirm the plasma viral load results, we assessed SIV expressing cells by *in situ* hybridization of peripheral lymph nodes sampled from AGM at one and four weeks p.i. (**Fig. 3C** and **4**). As shown in **Fig. 3C**, the number of SIV positive cells in lymph node biopsies from control or antibody-treated AGM at one or four weeks p.i. did not differ significantly (*P* = 0.486 and *P* = 0.183).

As reported previously [29], the kinetics of viremia in PTM and vervet AGM inoculated with SIV_agmVer90_ is similar. As expected, all control PTM experienced a peak of viremia at 7–10 days p.i. followed by a decline to setpoint viremia (**Fig. 3D**). In contrast to the AGM, we detected a significantly higher SIV plasma viral RNA copy number in CD8+ and CD20+ lymphocyte-depleted PTM than in control SIV infected PTM (median; lymphocyte-depleted: 8.79×10^8^ versus control: 0.66×10^8^, *P* = 0.029; **Fig. 3E**). Plasma viral load remained significantly higher throughout the remainder of their disease course (*P* = 0.029 at 3 and 6 weeks p.i.). The viral load in the inefficiently-depleted PTM (P24) decreased to a level seen at the high end of the range of the historic control PTM. The higher viral load in the lymphocyte-depleted PTM was confirmed by analysis of SIV expression in lymph node sections. As shown in **Fig. 3F** **and** **4**, there was a trend to higher levels of SIV+ cells in PTM lymph nodes collected at one week p.i. (*P* = 0.057) and a significantly higher number of SIV+ cells at four weeks p.i. (*P* = 0.029).

### CD8+ and CD20+ lymphocyte depletion affects survival of SIV-infected PTM but not AGM

**Figure 5:**
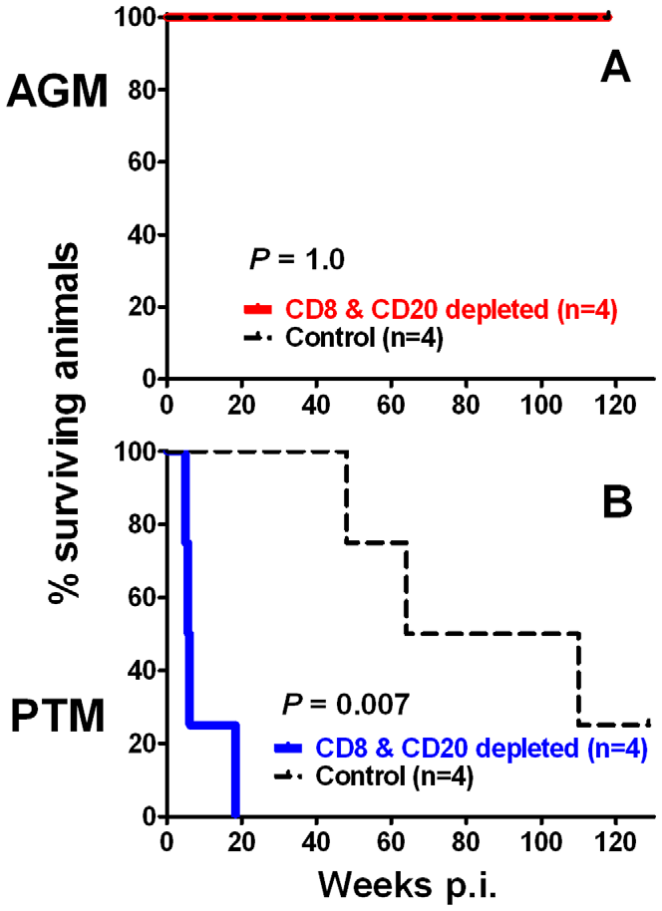
Reduced survival of SIV_agmVer90_-infected CD8+ and CD20+ lymphocyte-depleted PTM in contrast to disease-free survival of AGM. Kaplan-Meier survival curves for CD8+ and CD20+ lymphocyte-depleted and historic control vervet AGM (**A**) and PTM (**B**) are shown. Differences in survival were determined by log rank test.

All of the SIV-infected AGM remained healthy throughout the original observation period of 50 weeks p.i. and beyond despite efficient lymphocyte depletion in the mAb-treated group (**Fig. 5A**). In striking contrast to the AGM, CD8+ and CD20+ lymphocyte depletion in PTM resulted in an accelerated disease progression compared with historical control PTM infected with the same inoculum (**Fig. 5B**; log rank test; *P* = 0.007). Three of the four PTMs with almost complete lymphocyte depletion developed respiratory distress that necessitated euthanasia by six weeks p.i. The fourth animal had only a transient CD8+ and CD20+ lymphocyte depletion and experienced similar clinical signs during the primary stages of infection but made a partial clinical recovery. This animal subsequently experienced episodes of vasculitis associated with infarction of the skin, weight loss, poor appetite and diarrhea that eventually led to euthanasia at 18 weeks p.i. Pathologic evaluation of the three PTM euthanized early in the infection were remarkably similar, and included severe lymphoid depletion, severe vasculitis with pulmonary edema and cytomegalic cells in the lung, pulmonary edema, and nuclear inclusions consistent with disseminated cytomegalovirus (CMV) infection. SIV-specific *in situ* hybridization showed high levels of SIV-expression in all lymphoid tissues and in the brain or meninges consistent with uncontrolled SIV replication (data not shown).

### CD8+ and CD20+ lymphocyte depletion in the context of SIV infection induces massive CMV reactivation in PTM

**Figure 6:**
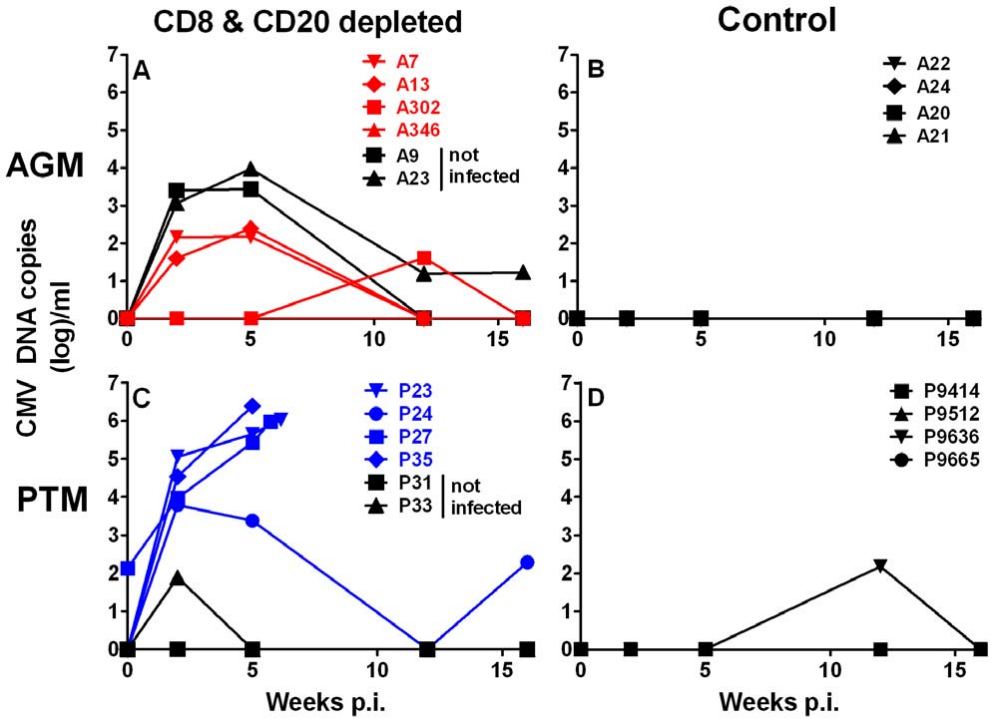
CD8+ and CD20+ lymphocyte depletion results in CMV reactivation in AGM and PTM. CMV DNA copies in plasma of vervet AGM (**A**) and PTM (**C**) are shown graphically for animals not infected with SIV_agm_ but depleted of CD8+ and CD20+ lymphocytes (black symbols), and animals that were depleted of CD8+ and CD20+ lymphocytes and SIV_agmVer90_-infected (red for vervet AGM, blue for PTM). For comparison, historic SIV-infected control AGM (**B**) and PTM (**D**) that did not receive administrations of mAbs are shown. The median values of six replicates for sequential plasma samples at each time point from each animal are shown.

To confirm the pathologic findings and to examine the effect of CD8+ and CD20+ lymphocyte depletion on CMV reactivation, we assayed CMV DNA in the plasma of both the AGM and PTM by quantitative PCR. As shown in **Fig. 6B and D**, SIV infection alone did not result in significant activation of CMV either in AGM or PTM. However, CMV activation was observed in the majority of the animals that were CD8+ and CD20+ lymphocyte-depleted as indicated by detectable CMV DNA in plasma (**Fig. 6A and C**) regardless of whether they were also infected with SIV. Reactivation of CMV was transient in the SIV infected and CD8+ and CD20+ lymphocyte-depleted AGM. In contrast, massive reactivation of CMV (up to 10^6^ copies/ml of plasma) was observed in three of the CD8+ and CD20+ lymphocyte depleted and SIV-inoculated PTM. The PTM with the shortest duration of CD8+ T cell and B cell depletion transiently controlled CMV replication by week 12 but CMV DNA in plasma was detectable again prior to death. Reactivation of CMV in lymphocyte-depleted monkeys shows that control of CMV is dependent on functional CD8+ and CD20+ lymphocytes, as observed in AGM. PTM however appeared to be too compromised either by high level SIV viremia and/or lack of CD8+ and CD20+ lymphocytes to mount an effective immune response against CMV.

### Massive loss of CD4+ T cell in SIV-infected PTM

**Figure 7:**
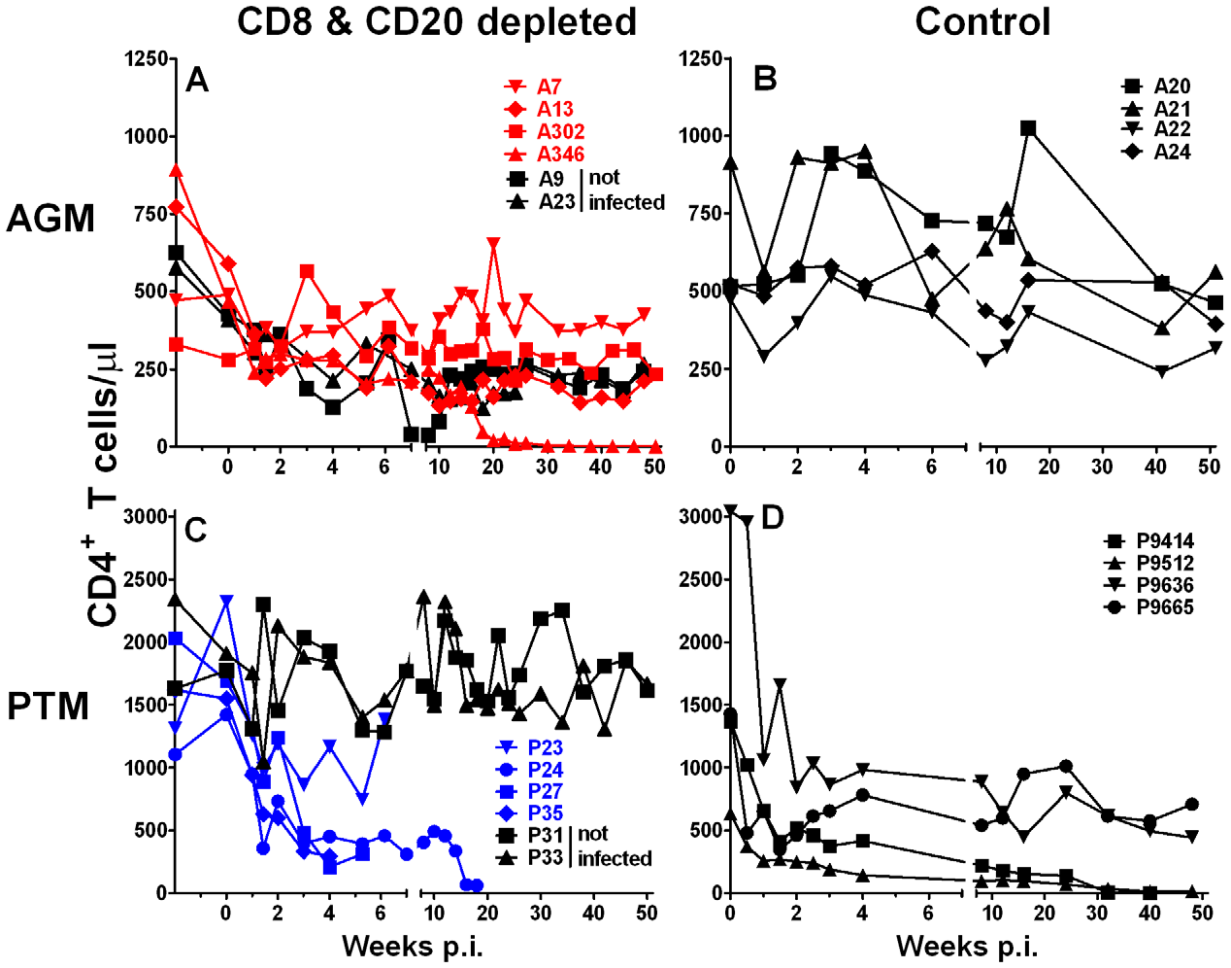
CD4+ T cell count changes in PTM and vervet AGM. Effect of CD8+ and CD20+ lymphocyte depletion on absolute CD4+ T cell counts in peripheral blood of vervet AGM (**A**) and PTM (**C**) that were inoculated with SIV_agmVer90_ (color symbols) or not infected (black symbols). Absolute CD4+ T cell counts in peripheral blood of historic control vervet AGM (**B**) and PTM (**D**) that were inoculated with the same dose of SIV_agmVer90_ but were not treated with mAbs are shown for comparison.

The majority of CD4+ T cells in vervet AGM coexpress the CD8αα homodimer [18]. Administration of the anti-CD8α mAb therefore had a potential depleting effect on CD4+ T cells. Absolute CD4+ T cell counts in the blood declined with a median of −46% (range: −59% to +6%) following administration of the anti-CD8α mAb, regardless of inoculation with SIV (**Fig. 7A**). Interestingly, CD4+ T cell levels did not return to pre-treatment values, but remained relatively stable in the AGM with exception of A346. This animal experienced an abrupt decline in peripheral blood CD4+ T cell numbers after week 14 that coincided with a slight increase in plasma viremia. Viremia subsequently decreased and the animal remained free from clinical signs of disease throughout one year of follow up, despite a very low frequency of CD4+ T cells (<5 cells/µl).

In contrast to the vervet AGM, CD4+ T cells in PTM do not express the CD8αα homodimer. Therefore, administration of the anti-CD8α antibody did not affect CD4+ T cell counts in the non-infected mAb treated PTM (**Fig. 7C**). The relatively stable number of peripheral blood CD4+ T cells in AGM was not observed for the lymphocyte-depleted PTM where SIV infection resulted in an abrupt decline in peripheral blood CD4+ T cells in the first 2 weeks of infection (**Fig. 7C**). CD4+ T cell levels continued to decline in three of the animals (P24, P27, and P35). PTM P23 showed a less dramatic loss of CD4+ T cells than the other three lymphocyte-depleted PTM (**Fig. 7C**). Similar kinetics and extent of CD4+ T cell decline were observed in untreated control PTM infected with SIV (**Fig. 7D**). CD4+ T cell decline was less severe in the two PTM with lower viremia (P9665, P9663). These two animals also showed the longest survival (>105 weeks) [13].

### Increased naive to memory CD4+ T cell ratio and CD4+ T cell proliferation in PTM but not AGM

**Figure 8:**
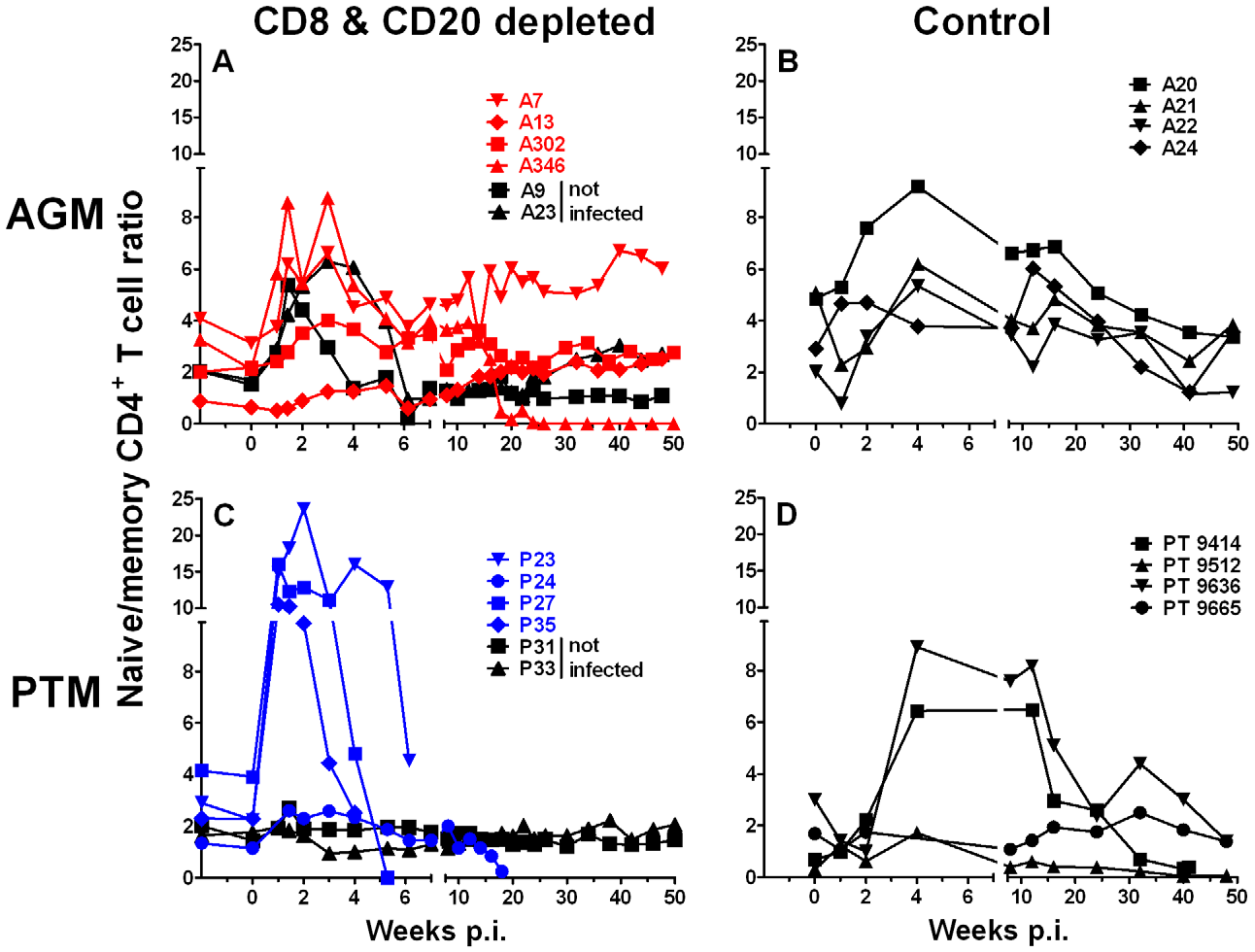
Increase in naive/memory CD4+ T cell ratio in SIV_agmVer90_-infected, efficiently CD8+ and CD20+ lymphocyte-depleted PTM. Ratio of naive to memory CD4+ T cells in peripheral blood of vervet AGM (**A**) and PTM (**C**). SIV_agmVer90_-challenged vervet AGM and PTM are shown with red and blue symbols, respectively. Animals that received the anti-CD8 and anti-CD20 depleting antibodies but were not inoculated with SIV are shown in black symbols. The ratio of naive to memory CD4+ T cells is shown for four control SIV-infected vervet AGM (**B**) and PTM (**D**) in black symbols and lines.

The decline of CD4+ T cells in pathogenic models of AIDS infected with a CCR5-tropic virus is mainly due to a loss of memory CD4+ T cells [6],[30]. To assess the extent of memory CD4+ T cell depletion, we evaluated the ratio of naïve to memory CD4+ T cells in PTM and AGM. We observed an initial loss of memory CD4+ T cells at peak viremia, as indicated by a higher ratio of naïve to memory CD4+ T cells than before infection (**Fig. 8A, B**) in all but one SIV-infected AGM. The non-infected lymphocyte-depleted AGM experienced a similar increase in the ratio of naïve to memory CD4+ T cells as SIV-infected AGM (**Fig. 8A**). In AGM, memory CD4+ T cells express higher levels of CD8α than naive CD4+ T cells (data not shown) and therefore may be preferentially depleted by the anti-CD8 mAb. After the transient loss of memory CD4+ T cells, the naïve to memory CD4+ T cell ratio recovered to levels comparable to pre-infection ratios, with the exception of A7. A7 recovered from the initial loss of memory CD4+ T cells to a higher, but nevertheless stable ratio of naïve to memory CD4+ T cells. As described above, one of the AGM (A346) suffered an almost complete loss all of its peripheral blood CD4+ T cells (**Fig. 7A**). The loss of CD4+ T cells also included a precipitous decline of naive CD4+ T cells as indicated by the decline in the naive to memory CD4+ T cell ratio indicated (**Fig. 8A**).

In contrast to the effects on the CD4+ T cell subset in vervet AGM, a much more obvious but also transient increase in the naive to memory CD4+ T cell ratio was observed in three of four CD8+ and CD20+ lymphocyte-depleted, SIV-infected PTM (**Fig. 8C**). The short term depleted PTM, P24, only showed a minor increase in the naïve to memory CD4+ T cell ratio with a subsequent slow decline to low levels just prior to death. In contrast, a much more muted decline in memory CD4+ T cells was observed in SIV-infected control PTM (**Fig. 8D**).

**Figure 9:**
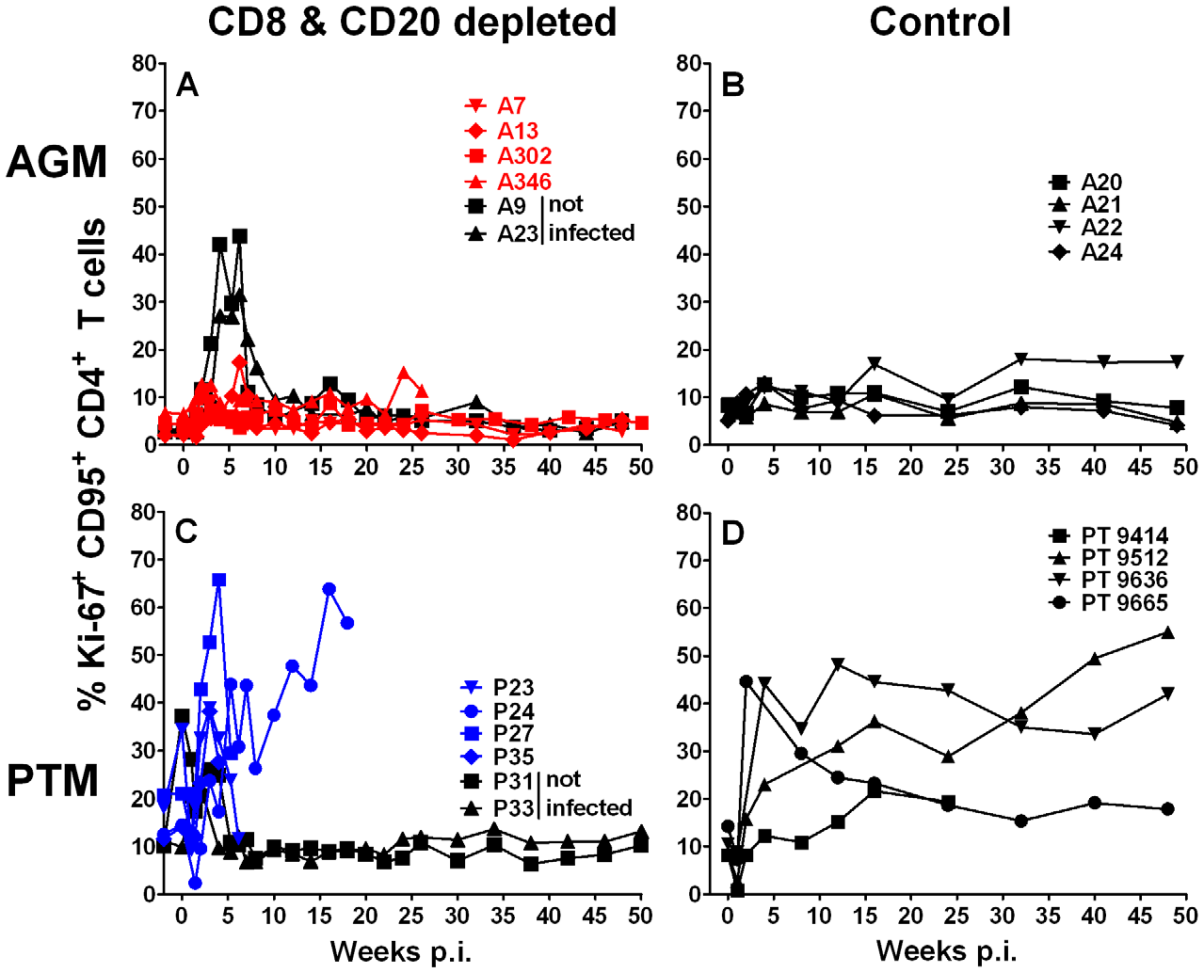
Ki-67 expression in memory CD4+ T cells from vervet AGM and PTM. Ki-67 expression of CD4+ T cells in peripheral blood of vervet AGM (**A**) and PTM (**C**). SIV_agmVer90_-challenged vervet AGM and PTM are shown with red and blue symbols respectively. Animals that received the anti-CD8 and anti-CD20 depleting antibodies but were not inoculated with SIV are shown in black symbols. For comparison, the percentage of Ki-67+ CD4+ T cells is shown for four control SIV-infected vervet AGM (**B**) and PTM (**D**) in black symbols and lines.

A more severe pathogenic SIV infection is generally associated with an increased turnover of CD4+ T cells. The increased proliferation of CD4+ T cells can be directly assessed by an increase in Ki-67 expression on memory CD4+ T cells. We therefore evaluated the proliferation of memory CD4+ T cells in both AGM and PTM (**Fig. 9**). As expected, a vigorous proliferation of memory CD4+ T cells was seen in both the lymphocyte-depleted and the historical control SIV-infected PTM (**Fig. 9C and D**). In contrast to the pathogenic SIV infection, we only observed a marginal increase in proliferation of memory CD4+ T cells in both of the two SIV-infected groups of AGM (**Fig. 9A and B**). The brief increase of memory CD4+ T cell proliferation in both SIV negative AGM and PTM was likely the result of compensatory homeostatic mechanisms in response to the depletion of CD8+ T cells (**Fig 9A and B**).

### B cell depletion induces a delay in SIV-specific antibody responses but has marginal effect on viremia

**Figure 10:**
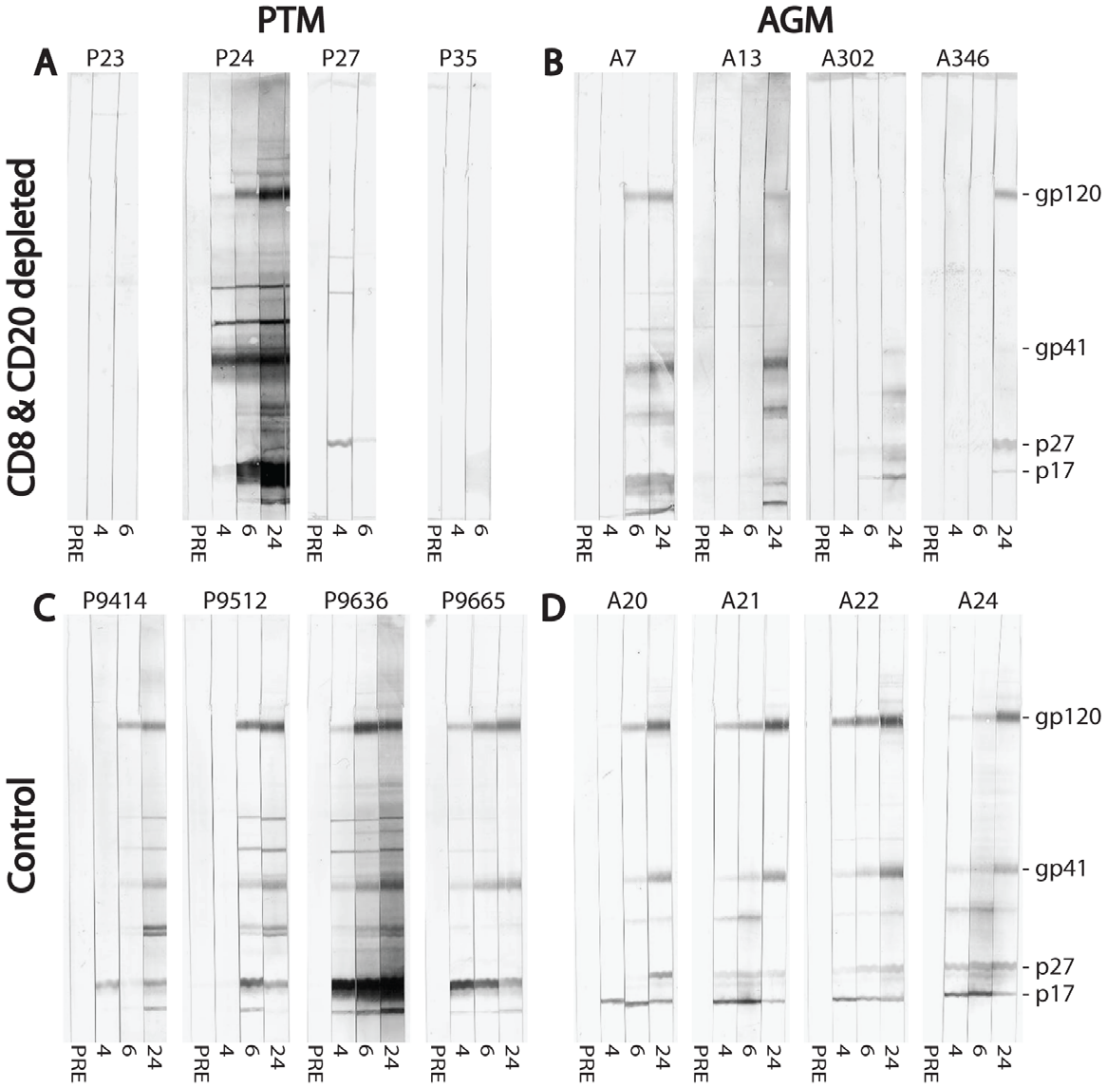
Impairment of SIV_agm_-specific antibodies in CD8+ and CD20+ lymphocyte-depleted vervet AGM and PTM. Development of SIV_agm_-specific antibody responses by Western blot analysis is shown for CD8+ and CD20+ lymphocyte-depleted vervet AGM (**A**) and PTM (**B**) in the top row. The bottom row shows historic SIV_agm_-infected vervet AGM (**C**) and PTM (**D**) as controls. Numbers below the strips indicate time in weeks following SIV_agmVer90_ infection. The location of the major SIV proteins is shown on the right.

Even low numbers of remaining B cells in lymphoid tissue after CD20+ lymphocyte depletion may support the generation of SIV-specific antibodies. We therefore evaluated the efficacy of the inhibition of humoral immune responses by determining the generation of SIV-specific antibody responses by Western blot and neutralization assays. Western blot assays of AGM plasma against whole SIV_agmVer90_ virus lysate revealed a delay in the development of SIV-specific antibodies in three of the four lymphocyte-depleted AGM compared to historical non-depleted control AGM inoculated with the same virus (**Fig. 10A and C**). All control AGM developed SIV-specific antibodies by four to six weeks p.i. Similarly the inefficiently CD20+ lymphocyte-depleted AGM (A7) developed SIV-specific antibodies by six weeks p.i. The three long-term B cell-depleted AGM only seroconverted after week six, and two animals showed only a weak response to SIV antigen even at 24 weeks post infection.

**Figure 11:**
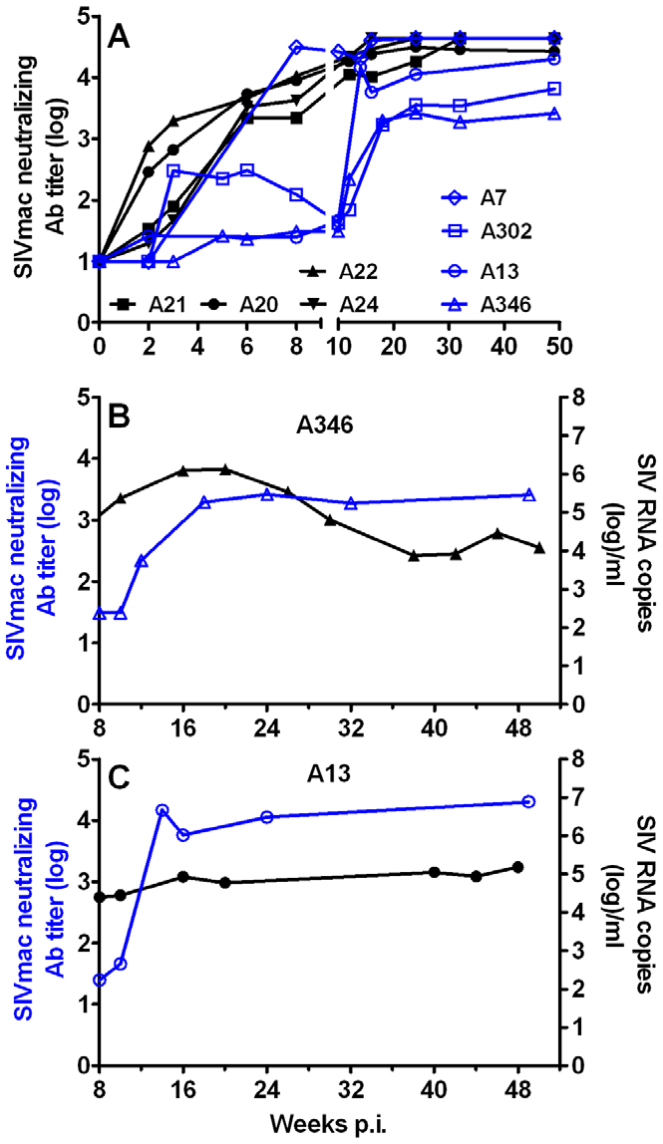
CD8+ and CD20+ lymphocyte depletion delays the generation of neutralizing antibodies in SIV_agmVer90_-infected AGM. A neutralizing antibody assay using tissue culture lab-adapted SIV_mac251_ was utilized to detect neutralizing Ab in historical control (black symbols) and CD8+ and CD20+ lymphocyte-depleted SIV_agmVer90_-infected vervet AGM (**A**). Impact of delayed appearance of neutralizing Ab on set point viremia in two long-term B cell-depleted animals (A346: **B**; A13: **C**).

A more dramatic effect on seroconversion was observed in PTM that were depleted of CD8+ T cells and CD20+ cells (**Fig. 10B**). Only the PTM (P24) transiently depleted of CD8+ T cells and B cells developed SIV-specific antibody responses at four weeks p.i. The three other B cell-depleted PTM did not develop any SIV specific antibodies. All of the historic control PTM developed variable levels of SIV-specific antibody responses by four to six weeks p.i. (**Fig. 10D**). We attempted to confirm these data using SIV_agmVer90_ envelope-pseudotyped HIV particles in a single round neutralizing antibody (Ab) assay [31]. However, SIV_agmVer90_ appeared to be highly resistant to neutralization and therefore we were unable to detect neutralizing Ab in any of the AGM in this study. However, using a tissue culture lab-adapted SIV_mac251_ strain, we observed a delay in the generation of neutralizing Ab in AGM that had been well-depleted of B cells compared to the historic non-depleted SIV infected control AGM (**Fig. 11A**). The appearance of neutralizing Ab had no consistent effect on the magnitude of plasma viremia in either of these AGM (**Fig. 11B and C**).

## Discussion

Soon after the discovery of AIDS viruses it became apparent that natural hosts of SIV do not generally develop immunodeficiency in association with SIV infection, whereas these viruses readily induce disease in Asian nonhuman primates and humans [8],[12],[32],[33],[34]. However, the mechanisms employed by these primates to avoid the pathogenic consequences of SIV-infection remain unclear.

In the present study, we made a direct comparison of the role of SIV-specific adaptive immune responses in a nonnatural host and natural host of SIV infection, PTM and vervet AGM, respectively. To do this, we evaluated the effect of antibody-mediated temporal inhibition of cellular and humoral immune responses during primary infection with the uncloned SIV virus, SIV_agmVer90_, in PTM and AGM. Recent investigations have shown that this virus does not induce an AIDS-like disease in AGM but is not inherently nonpathogenic as infection studies in PTM have shown [25]. Here in this study, temporal inhibition of adaptive immune responses in primary SIV infection of PTM resulted in an increased peak and set point viremia and accelerated disease progression similar to observations recently made in CD8+ lymphocyte-depleted rhesus macaques infected with pathogenic SIV [35],[36],[37]. Interestingly, in the present study and in unpublished observations evaluating sabaeus AGM (R.C. Zahn et al.), peak viremia was not increased in lymphocyte-depleted AGM but only a relatively brief prolongation of peak viremia was observed during primary infection. The delay in resolution of primary viremia was very likely due to the inhibition of cellular immune responses since primary viremia had resolved before the appearance of humoral immune responses. Also, the eventual generation of humoral immune responses in B cell-depleted animals following reappearance of these cells did not appear to have a significant influence on the magnitude of viremia. This observation was recently confirmed by others when B cell depletion during primary and chronic SIV infection of AGM did not result in an increased viremia or clinical signs of illness (personal communications with Ivona Pandrea). Thus, the data presented here and unpublished observations using sabaeus AGM (R.C. Zahn et al.) suggest that cellular immune responses contribute to viral containment in AGM but humoral immune responses appear to be less critical. However, in contrast to rhesus monkeys the absence of CD8+ lymphocytes in AGM resulted in a much more subdued impact on viremia, similar to observations recently made in sooty mangabeys [38].

Although no overt signs of SIV disease were seen in the lymphocyte-depleted AGM, the depletion of these cells was associated with reactivation of CMV. A similar transient reactivation of CMV without clinical signs was observed in one of the two PTM that were lymphocyte-depleted but not challenged with SIV. The most significant signs of pathogenicity were seen in PTM that were depleted of CD8+ and CD20+ lymphocytes and inoculated with SIV: in these animals, we observed a massive increase in plasma CMV DNA copies, precipitous loss of CD4+ T cells and an increase in naive/memory CD4+ T cell ratio, indicating a rapid loss of memory CD4+ T cells.

These observations raise a number of questions. Is it possible that the impairment of adaptive immune responses in AGM was not of sufficient duration to negatively impact the health of these animals? Or, is the inherent ability to suppress immune activation in SIV-infected AGM the critical factor that helps AGM to cope with chronic SIV infection?

CD8+ lymphocyte depletion in rhesus macaques results in a significantly enhanced disease progression. However, as recently shown [35],[39],[40] and observed here in the PTM depleted of CD8+ lymphocytes, the fastest disease acceleration is seen in nonnatural hosts when CD8+ lymphocytes are depleted for at least the first 4 weeks during primary SIV infection. However, the CD8+ lymphocyte depletion in most AGM in this study was of fairly short duration. But even the one relatively long-term CD8+ lymphocyte-depleted AGM (A13; about 6 weeks) did not develop an AIDS like-disease with rapid disease progression as we have seen in all rhesus macaques studied so far with a similar length of CD8+ lymphocyte-depletion [39],[40]. In addition, PTM P24 in this study also was only depleted for 2 weeks, but succumbed to AIDS within 18 weeks p.i.

A number of recent investigations have shown that natural hosts exhibit a much lower level of immune activation during chronic viremia compared to nonnatural hosts [16],[32]. The low level immune activation may protect the natural host species from more aggressive virus replication and the development of an AIDS-like disease. Recent investigations have shown that short-term immune activation using LPS or an IL-2/diphtheria toxin fusion protein in AGM can result in an increased viremia, supporting the notion that hyperactivation of the immune system plays a role in virus replication and disease progression [41]. However, these brief *in vivo* manipulations had no apparent effect on the health of the animals. Thus, *in vivo* manipulations in AGM that are capable of suppressing adaptive immune responses for a longer duration than in the present study and/or induce a prolonged immune activation may result in a different outcome.

In addition, there may be a number of possible caveats in our study. (1). Since CD4+ T cells in vervet AGM dimly coexpress the CD8α molecule, administration of the anti-CD8α antibody may have also affected CD4+ T cell targets and thus limited virus replication. However, a similar result was recently observed in sabaeus AGM that did not coexpress the CD8α molecule on CD4+ T cells (R.C. Zahn et al., unpublished observations). We also cannot clearly rule out that NK cells that express the CD8α molecule may contribute to viral containment as these cells are also depleted by the anti-CD8α antibody. It is also possible that some of the differences observed in the control group and the antibody-treated group may be due to utilizing historical controls for this study. However, both the historical controls and antibody-treated group were treated identically except for receiving lymphocyte-depleting antibodies. Finally, it is conceivable that the combined depletion of CD8+ and CD20+ lymphocytes may affect AGM and PTM differently. However, to formally rule out that the antibody treatment may have a more significant pathogenic effect on PTM we have performed the lymphocyte depletion experiments as well in SIV-negative AGM and PTM which both did not exhibit any signs of disease following the antibody administrations.

Recent investigations have shown that evolutionary adaptations in natural hosts (paucity of CCR5+ cells, decreased immune activation, and ability to down modulate the CD4 molecule on CD4+ T cells) may assist adaptive immune responses or may even render SIV-specific adaptive immune responses unnecessary [32],[42],[43]. In contrast, if the AIDS virus can bypass restriction factors in nonnatural hosts, adaptive immune responses appear to be the major defense against uncontrolled virus replication. However, the eventual failure of viral control is due to inevitable viral immune escape [44],[45]. A sign of the incredible plasticity of the immune system of natural hosts to cope with SIV infection was seen in one of the lymphocyte-depleted AGM (A346). This animal eventually lost all of its peripheral blood CD4+ T cells (both memory and naïve cells), suggesting that the virus in this animal might have changed coreceptor usage (characterization of coreceptor usage is still ongoing). Even with an almost complete loss of peripheral blood CD4+ T cells the animal showed no signs of disease. Recently, a similarly abrupt decline in CD4+ T cells was observed in another natural host of SIV, sooty mangabeys, upon emergence of a CXCR4-tropic SIV variant without inducing disease [46]. This abrupt decline of CD4+ T cells in sooty mangabeys does not necessarily always depend on switching the tropism of the virus to CXCR4 [47]. AGM may be capable of utilizing a large fraction of CD4- T cells, which can be found in peripheral blood and tissues, as surrogate T helper cells [43],[48].

Investigations into natural hosts of SIV, including AGM, will allow us to understand how these animals can coexist with SIV without developing disease. The observations made here and in sabaeus AGM (R.C. Zahn et al., unpublished observations) suggest that CD8+ T cell responses participate to some degree in controlling viral replication in natural hosts. However, the effects were considerably more limited than observations made in macaques and it is not clear whether a more long-term increase in viremia would precipitate disease progression in AGM. Further investigations are required to assess the relative contribution of adaptive immune responses versus non-adaptive mechanisms in the maintenance of an AIDS-free course of infection in natural host species. Our aim is that these investigations will provide clues how pathogenic AIDS virus infections could be limited, identify new therapeutic approaches, and contribute to the development of a successful HIV vaccine.

## Materials and Methods

### Ethics statement

All animals were maintained in accordance with the guidelines of the Committee on the Care and Use of Laboratory Animals under a NIAID-approved animal study protocol [49], and all studies and procedures were reviewed and approved by the Institutional Animal Care and Use Committees of the NIH and Harvard Medical School.

### Animals and viruses

Animals were inoculated intravenously with SIV_agmVer90_, an isolate from a naturally-infected vervet AGM (*Chlorocebus pygerythrus*) imported from Kenya in 1987 [7]. The virus (SIV_agmVer90_) was isolated from the mesenteric lymph nodes of monkey AGM90 by coculture of viably frozen mononuclear cells with pigtailed macaque peripheral blood mononuclear cells (PBMC). The vervet AGM utilized for this study were imported from Tanzania and screened for SIV infection by Western blotting, virus isolation from PBMC, and plasma viral RNA (vRNA) loads. PTM were colony bred in North America. All study animals were seronegative for SIV, respiratory syncytial virus (SRV), and simian T-cell leukemia virus (STLV-1).

### Animal study design

A total of six adult vervet AGM and six adult PTM were recruited for combined CD8+ and CD20+ lymphocyte depletion studies. The chimeric anti-human CD8α monoclonal antibody (mAb), cM-T807 (NIH Nonhuman Primate Reagent Resource) was administered at 10 mg/kg of body weight subcutaneously on day 0 (the day of SIV infection) followed by 5 mg/kg intravenous injections on days 3, 7, 10 and 14. The anti-human CD20 mAb, Rituxan® (Rituximab), purchased from Genentech, Inc. (South San Francisco, CA), was administered intravenously at 50 mg/kg of body weight on days −7, 14 and 35. For lymph node biopsies, animals were sedated with Telazol®. For all other procedures including brochoalveolar lavage (BAL), phlebotomy and Ab injections, animals were sedated with ketamine hydrochloride. Of these twelve treated animals, four AGM and four PTM were inoculated intravenously with 1,000 50% tissue culture infectious doses (TCID_50_) of SIV_agmVer90_ on day 0. The remaining antibody-treated animals (two AGM and two PTM) were not infected. Animals were monitored for 50 weeks following inoculation by plasma viral load, SIV-specific antibody responses by Western blot, lymphocyte subsets in the blood, BAL and lymph node biopsies (−2, 1, and 4 weeks p.i.), and clinical evidence of disease. Animals showing weight loss of greater than 10% of body weight, diarrhea, or evidence of pneumonia that was unresponsive to antibiotic or supportive therapy were humanely euthanized and tissues collected for pathology. An additional four AGM and four PTM previously inoculated with the same SIV_agmVer90_ stock and dose served as historic, untreated controls [7],[29].

### Plasma viral load assay

Plasma levels of viral SIV RNA in PTM and AGM were measured by a quantitative real-time RT-PCR assay as previously described [7], using methodology based on the 7700 sequence detection system (Applied Biosystems, Foster City, CA). Plasma samples were collected from EDTA-anticoagulant whole blood and were stored in a −80°C freezer until analysis. Plasma viral RNA was isolated using a QIAmp viral RNA kit (QIAGEN, Valencia, CA), and RT-PCR reactions were performed in 96-well plates.

### Measurement of CMV DNA in plasma by real-time PCR

CMV DNA was quantitated in plasma using real-time PCR as previously described [50]. DNA was extracted from plasma with the QIAmp DNA Mini Kit (Qiagen, Inc., Valencia, CA). The rhesus CMV specific primers amplify a 108-bp amplicon in the exon 1 region of the immediate-early gene of rhesus CMV [51] and are reactive with the published AGM CMV [52]. The forward primer (5-GTTTAGGGAACCGCCATTCTG-3) corresponds to residues 4847 to 4867 of AGM CMV, the reverse primer (5-GTATCCGCGTTCCAATGCA-3) corresponds to residues 4936 to 4954, and the probe (5-FAM-TCCAGCCTCCATAGCCGGGAAGG-tamra-3) corresponds to residues 4908 to 4930. The PCR was run on an ABI-Prism 7700 Sequence Detection System (PerkinElmer, Foster City, CA) [50].

### Immunohistochemistry and SIV-specific *in situ* hybridization

Nonradioactive *in situ* hybridization (ISH) for SIV expression was performed in formalin-fixed, paraffin-embedded lymph nodes utilizing sense or antisense digoxigenin labeled riboprobes that spanned the entire SIV_agm9063-2_ genome as previously described [25]. The number of SIV-expressing cells was measured as follows: Six random fields of view were selected for each of the ISH stained lymph nodes and the AxioVision automated segmentation measurement program was used to calculate the number of SIV+ cells per high powered field (HPF). Lymph node biopsies were also evaluated for CD20 positive cells by using a mouse anti-human CD20 antibody (M0755, DAKO Cytomation, Carpenteria, CA), a mouse IgG avidin biotin complex-peroxidase kit (Vector Laboratories, Ltd., Burlingame, CA), and diaminobenzidine (DAB) substrate.

### Western blot analysis for SIV-specific antibodies

Serology for antibodies to SIV_agm_ was performed by Western blot analysis, as previously described [25]. Briefly, virus was pelleted from cell-free supernatant of CEMss cells infected with SIV_agmVer90_. Virus particles were disrupted in Laemmli sample buffer, viral proteins were separated by SDS/polyacrylamide gel electophoresis and transferred onto nitrocellulose membranes. Individual strips containing SIV_agm_ viral proteins were reacted with diluted PTM and AGM plasma and washed to remove unbound material. The bound SIV specific antibodies were visualized by subsequent reaction with ImmunoPure A/G protein conjugated with alkaline phosphatase (Pierce Biotechnology, Rockford, IL), followed by nitroblue tetrazolium-5-bromo-4-chloro-3-indolylphosphate (BCIP/NBT) substrate system (KPL, Laboratories, Gaithersburg, MD).

### Serum neutralizing antibody assays

Neutralization was measured as a function of reduction in luciferase reporter gene expression after a single round of infection in TZM-bl cells as described [31]. TZM-bl cells were obtained from the NIH AIDS Research and Reference Reagent Program, as contributed by John Kappes and Xiaoyun Wu. Briefly, 200 TCID_50_ of virus was incubated with a serial 3-fold dilution of test sample in duplicates in a total volume of 150 µl for 1 h at 37°C in 96-well flat-bottom culture plates. Freshly trypsinized cells (10,000 cells in 100 µl of growth medium containing 75 µg/ml DEAE dextran) were added to each well. One set of control wells received cells and virus (virus control) and another set received cells only (background control). After a 48 h incubation, 100 µl of cells were transferred to a 96-well black solid plate (Corning, Lowell, MA) for measurement of luminescence using the Britelite Luminescence Reporter Gene Assay System (PerkinElmer). Neutralization titers are the dilution at which relative luminescence units (RLU) were reduced by 50% compared to virus control wells after subtraction of background RLUs. Assay stocks of molecularly cloned Env-pseudotyped viruses were prepared by transfection in 293T cells and were titrated in TZM-bl cells as described [31].

### Monoclonal antibodies and immunophenotyping of lymphocytes

All antibodies were purchased from BD Biosciences (San Jose, CA), BD Pharmingen (San Jose, CA), Caltag (Carlsbad, CA), R&D Systems (Minneapolis, MN) or Beckman Coulter (Miami, FL). The antibodies used in this study were anti-CD95-Allophycocyanin (DX2; BD Pharmingen), anti-CD28-PerCP-Cy5.5 (L293; BD Biosciences), anti-CD4-AmCyan (L200; BD Biosciences), anti-CD3-Pacific blue (SP34-2; BD Biosciences), anti-CD3-Alexa Fluor 700 (SP34; BD Pharmingen), anti-CD8α-Allophycocyanin-Cy7 (SK1; BD Biosciences), anti-CD8α-Phycoerythrin (DK25; Dako, Carpenteria, CA), anti-CD8α-PerCP-Cy5.5 (SK1; BD Biosciences), anti-CD8α-Allophycocyanin (SK1; BD Biosciences), anti-CD8αβ-Energy-Coupled Dye (ECD) (2ST8.5H7; Beckman Coulter), anti-CD20-Allophycocyanin-Cy7 (L27; BD Biosciences), CD79a-PerCP-Cy5.5 (HM47; BD Pharmingen), CD20-Phycoerythrin-Cy7 (L27; BD Biosciences), Ki-67-Fluorescein Isothiocyanate (B56; BD Biosciences). In order to determine the efficacy of lymphocyte depletion, whole blood was stained with anti-CD3, anti-CD4, anti-CD8, anti-CD8αβ, anti-CD20, and anti-CD79a antibodies. The use of the anti-CD8 clone DK25 (coupled to PE) and anti-CD79a permits detection of CD8+ lymphocytes and B cells with the best sensitivity in lymphocyte-depleted animals treated with the antibodies cM-T807 and Rituximab [53],[54]. For detection of maturation-associated T cell subsets, whole blood samples were stained for 15 min with anti-surface antibodies (CD3, CD4, CD8α, CD8αβ, CD28 and CD95). Red blood cells were lysed by a TQ-prep instrument (Beckman Coulter) and the cells were washed with PBS. For determination of proliferation, cells were then fixed and permeabilized with Cytofix/Cytoperm solution (BD Biosciences) according to the manufacturer's description and stained with anti-Ki-67 mAb. Labeled cells were fixed in 1.5% formaldehyde-phosphate-buffered saline. Samples were collected on an LSR II instrument (BD Biosciences) and analyzed using FlowJo software (TreeStar Inc., Ashland, OR). Mononuclear cells were purified from BAL samples and lymph node biopsies and viably frozen in media consisting of 20% DMSO and 80% FCS, and flow cytometry was performed on thawed suspensions.

### Statistical analyses

Statistical analyses and graphical presentations were computed with GraphPad Prism 5.02 (GraphPad Prism Software, La Jolla, CA). *P* values of <0.05 were considered significant. Mann-Whitney tests were applied for comparison of two groups. Kaplan-Meier graphs were used to compare survival, and log-rank tests were applied for statistical comparison.
